# 4-Methyl-*N*-(4-nitro­benzyl­idene)piperazin-1-amine

**DOI:** 10.1107/S1600536813028493

**Published:** 2013-10-23

**Authors:** Channappa N. Kavitha, Jerry P. Jasinski, Brian J. Anderson, H.S. Yathirajan, Manpreet Kaur

**Affiliations:** a Department of Studies in Chemistry, University of Mysore, Manasagangotri, Mysore 570 006, India; b Department of Chemistry, Keene State College, 229 Main Street, Keene, NH 03435-2001, USA

## Abstract

In the title compound, C_12_H_16_N_4_O_2_, the piperazine ring is in a slightly distorted chair conformation. In the mol­ecule, the mean plane of the nitro group is twisted by 8.0 (3)° from that of the benzene ring. Also, the mean plane of the 2-nitro­benzyl ring is twisted slightly from that of the piperazine ring, with an N—N=C—C torsion angle of −176.24 (11)°. In the crystal, pairs of weak C—H⋯O inter­actions link the mol­ecules into dimers approximately along [010].

## Related literature

For the biological activity of Schiff base piperzine derivatives, see: Kharb *et al.* (2012 ▶); Savaliya *et al.* (2010 ▶); Xu *et al.* (2009 ▶); Zhou *et al.* (2011 ▶). For therapeutic areas related to piperazines as drug mol­ecules, see: Bogatcheva *et al.* (2006 ▶); Brockunier *et al.* (2004 ▶); Cai *et al.* (2009 ▶); Choudhary *et al.* (2006 ▶); Upadhayaya *et al.* (2004 ▶). For a review of current pharmacological and toxicological information for piperazine derivatives, see: Elliott (2011 ▶). For the synthesis of related piperazine compounds and their medicinal and pharmaceutical activity, see: Capuano *et al.* (2002 ▶); Contreras *et al.* (2001 ▶). For related structures, see: Guo (2007 ▶); Ming-Lin *et al.* (2007 ▶); Xu *et al.* (2012 ▶); Zhou *et al.* (2011 ▶). For puckering parameters, see: Cremer & Pople (1975 ▶). For standard bond lengths, see: Allen *et al.* (1987 ▶).
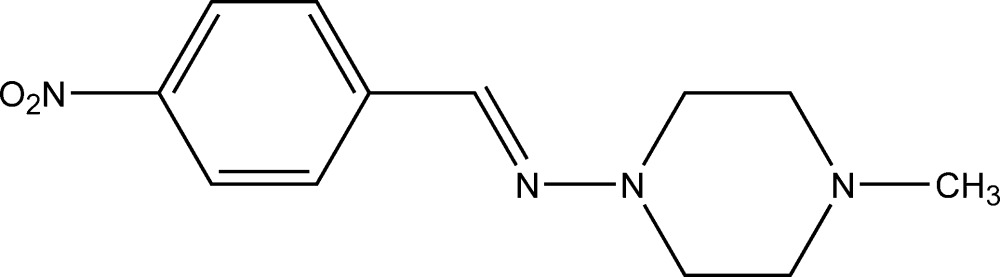


## Experimental

### 

#### Crystal data

C_12_H_16_N_4_O_2_
*M* *_r_* = 248.29
Monoclinic, 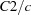

*a* = 27.9353 (14) Å
*b* = 5.9247 (3) Å
*c* = 18.7763 (7) Å
β = 126.527 (3)°
*V* = 2497.2 (2) Å^3^
*Z* = 8
Cu *K*α radiation
μ = 0.77 mm^−1^
*T* = 173 K
0.38 × 0.32 × 0.22 mm

#### Data collection

Agilent Xcalibur (Eos, Gemini) diffractometer
Absorption correction: multi-scan (*CrysAlis PRO* and *CrysAlis RED*; Agilent, 2012 ▶) *T*_min_ = 0.868, *T*_max_ = 1.000
7200 measured reflections
2439 independent reflections
2022 reflections with *I* > 2σ(*I*)
*R*_int_ = 0.031

#### Refinement

*R*[*F* ^2^ > 2σ(*F* ^2^)] = 0.041
*wR*(*F* ^2^) = 0.119
*S* = 1.02
2439 reflections
165 parameters
H-atom parameters constrained
Δρ_max_ = 0.22 e Å^−3^
Δρ_min_ = −0.18 e Å^−3^

### 

Data collection: *CrysAlis PRO* (Agilent, 2012 ▶); cell refinement: *CrysAlis PRO*; data reduction: *CrysAlis RED* (Agilent, 2012 ▶); program(s) used to solve structure: *SUPERFLIP* (Palatinus & Chapuis, 2007 ▶); program(s) used to refine structure: *SHELXL2012* (Sheldrick, 2008 ▶); molecular graphics: *OLEX2* (Dolomanov *et al.*, 2009 ▶); software used to prepare material for publication: *OLEX2*.

## Supplementary Material

Crystal structure: contains datablock(s) I. DOI: 10.1107/S1600536813028493/zl2568sup1.cif

Structure factors: contains datablock(s) I. DOI: 10.1107/S1600536813028493/zl2568Isup2.hkl

Supplementary material file. DOI: 10.1107/S1600536813028493/zl2568Isup3.cml

Additional supplementary materials:  crystallographic information; 3D view; checkCIF report

## Figures and Tables

**Table 1:** Hydrogen-bond geometry (Å, °)

*D*—H⋯*A*	*D*—H	H⋯*A*	*D*⋯*A*	*D*—H⋯*A*
C2—H2*B*⋯O1^i^	0.99	2.47	3.4052 (19)	158

Symmetry code: (i) 
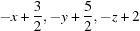
.

**Fig. 1.**
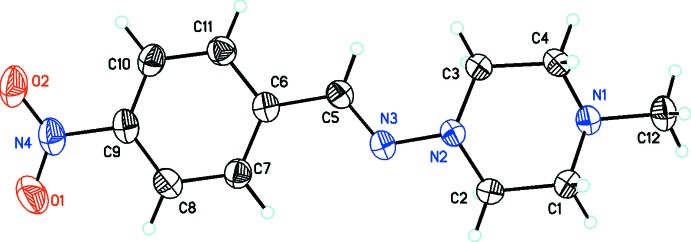
ORTEP drawing of (I) (C12H16N4O2 ) showing the labeling scheme with 50% probability displacement ellipsoids.

**Fig. 2.**
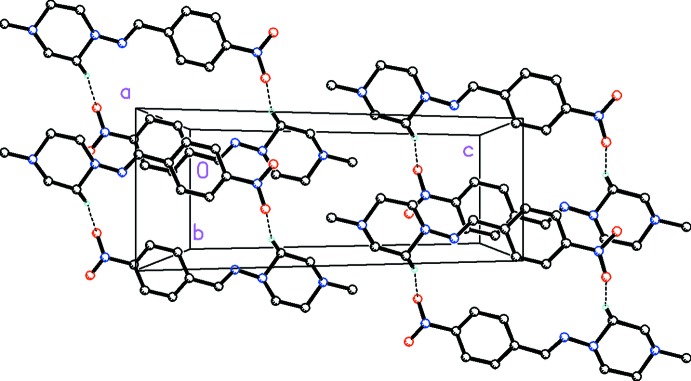
Molecular packing for (I) viewed along the a axis. Dashed lines indicate weak C—H···O intermolecular intereactions linking the molecules into dimers along [0 1 0]. H atoms not involved in hydrogen bonding have been removed for clarity.

## supplementary crystallographic information

### 1. Comment

Schiff base ligands derived from 1-amino-4-methylpiperazine have attracted interest due to diverse biological activities associated with the piperazine moiety. Schiff base piperazine derivatives have been designed to study their antimicrobial (Savaliya *et al.*, 2010; Kharb *et al.*, 2012)) and antibacterial activity (Xu *et al.*, 2012). In addition, many drugs contain a piperazine ring as part of their molecular structure (Cai *et al.*, 2009). Piperazines are among the most important building blocks in today's drug discovery and are found in biologically active compounds across a number of different therapeutic areas (Brockunier *et al.*, 2004; Bogatcheva *et al.*, 2006) such as antifungal (Upadhayaya *et al.*, 2004), anti-bacterial, antimalarial activity and as in antipsychotic agents (Choudhary *et al.*, 2006). A review on the current pharmacological and toxicological information for piperazine derivatives has been recently presented (Elliott, 2011). The synthesis of related piperazine compounds and their medicinal and pharmaceutical activity have also been reported (Contreras *et al.*, 2001; Capuano *et al.*, 2002). The crystal structures of some related compounds, viz., 2-[(4-methylpiperazin-1-yl)iminomethyl]phenol (Guo, 2007), 1,4-bis{3-[4-(dimethylamino)benzylideneamino] propyl}piperazine (Xu *et al.*, 2009), 2-methoxy-4-[(4-methylpiperazin-1-yl)- iminomethyl]phenol (Zhou *et al.*, 2011) and 2,4-dibromo-6- [(4-methylpiperazin-1-yl)iminomethyl]phenol (Ming-Lin *et al.*, 2007) have been reported. In view of the above importance of N-piperazinyl Schiff bases, the title compound, (I), C_12_H_16_N_4_O_2_ has been synthesized and the crystal structure is reported herin.

In the title compound, (I), the piperazine ring is in a slightly distorted chair conformation with puckering parameters Q, θ, and φ = 0.5646Å, 170.8 (5)° and 187.961 (8)° (Cremer & Pople, 1975) (Fig. 1). In the molecule, the mean plane of the nitro group is twisted by 8.0 (3)° from that of the phenyl ring. Also, the mean plane of the 2-nitrobenzyl ring is twisted slightly from that of the piperazine ring with an N1/N2/C5/C6 torsion angle of -176.24 (11)°. Bond lengths are in normal ranges (Allen *et al.*, 1987). Weak C—H···O intermolecular interactions are observed which lead to formation of dimers approximately along [010] and influence crystal packing (Fig. 2).

### 2. Experimental

To a solution of o-nitrobenzaldehyde (0.75 g, 0.005 mol) in 10 ml of methanol, an equimolar amount of (1-amino-4-methyl)piperazine (0.57 g, 0.005 mol) is added dropwise with constant stirring. The mixture was refluxed for 8 hours to obtain an orange solution. The solution was evaporated to a small volume at room temperature and allowed to stand. Yellow crystals were formed in one day (m.p.: 358–360 K) and were used as such for x-ray diffraction studies.

### 3. Refinement

All of the H atoms were placed in their calculated positions and then refined using the riding model with Atom—H lengths of 0.95Å (CH), 0.99Å (CH_2_) or 0.98Å (CH_3_). Isotropic displacement parameters for these atoms were set to 1.2 (CH, CH_2_) or 1.5 (CH_3_) times *U*_eq_ of the parent atom. Idealised Me were refined as rotating groups.

### Crystal data

**Table:**

C_12_H_16_N_4_O_2_	*F*(000) = 1056
*M**_r_* = 248.29	*D*_x_ = 1.321 Mg m^−^^3^
Monoclinic, *C*2/*c*	Cu *K*α radiation, λ = 1.54184 Å
*a* = 27.9353 (14) Å	Cell parameters from 2934 reflections
*b* = 5.9247 (3) Å	θ = 3.2–72.3°
*c* = 18.7763 (7) Å	µ = 0.77 mm^−^^1^
β = 126.527 (3)°	*T* = 173 K
*V* = 2497.2 (2) Å^3^	Irregular, yellow
*Z* = 8	0.38 × 0.32 × 0.22 mm

### Data collection

**Table:**

Agilent Xcalibur (Eos, Gemini) diffractometer	2439 independent reflections
Radiation source: Enhance (Cu) X-ray Source	2022 reflections with *I* > 2σ(*I*)
Detector resolution: 16.0416 pixels mm^-1^	*R*_int_ = 0.031
ω scans	θ_max_ = 72.3°, θ_min_ = 3.9°
Absorption correction: multi-scan (*CrysAlis PRO* and *CrysAlis RED*; Agilent, 2012)	*h* = −34→32
*T*_min_ = 0.868, *T*_max_ = 1.000	*k* = −7→7
7200 measured reflections	*l* = −15→22

### Refinement

**Table:**

Refinement on *F*^2^	Hydrogen site location: inferred from neighbouring sites
Least-squares matrix: full	H-atom parameters constrained
*R*[*F*^2^ > 2σ(*F*^2^)] = 0.041	*w* = 1/[σ^2^(*F*_o_^2^) + (0.0636*P*)^2^ + 0.9105*P*] where *P* = (*F*_o_^2^ + 2*F*_c_^2^)/3
*wR*(*F*^2^) = 0.119	(Δ/σ)_max_ = 0.001
*S* = 1.02	Δρ_max_ = 0.22 e Å^−^^3^
2439 reflections	Δρ_min_ = −0.18 e Å^−^^3^
165 parameters	Extinction correction: *SHELXL2012* (Sheldrick, 2008), Fc^*^=kFc[1+0.001xFc^2^λ^3^/sin(2θ)]^-1/4^
0 restraints	Extinction coefficient: 0.00056 (10)
Primary atom site location: structure-invariant direct methods	

### Special details

**Table:**

Geometry. All esds (except the esd in the dihedral angle between two l.s. planes) are estimated using the full covariance matrix. The cell esds are taken into account individually in the estimation of esds in distances, angles and torsion angles; correlations between esds in cell parameters are only used when they are defined by crystal symmetry. An approximate (isotropic) treatment of cell esds is used for estimating esds involving l.s. planes.

### Fractional atomic coordinates and isotropic or equivalent isotropic displacement parameters (Å^2^)

**Table:**

	*x*	*y*	*z*	*U*_iso_*/*U*_eq_	
O1	0.93562 (6)	1.1504 (2)	1.26970 (8)	0.0636 (4)	
O2	0.96924 (5)	0.8095 (2)	1.29508 (7)	0.0509 (3)	
N1	0.60437 (5)	0.7685 (2)	0.55865 (7)	0.0301 (3)	
N2	0.66959 (5)	0.80238 (19)	0.74619 (7)	0.0282 (3)	
N3	0.71366 (5)	0.8675 (2)	0.83118 (7)	0.0290 (3)	
N4	0.93437 (5)	0.9567 (3)	1.24576 (8)	0.0403 (3)	
C1	0.58784 (6)	0.9349 (2)	0.59774 (9)	0.0328 (3)	
H1A	0.5570	0.8709	0.6023	0.039*	
H1B	0.5710	1.0702	0.5594	0.039*	
C2	0.64208 (6)	1.0007 (2)	0.68894 (9)	0.0329 (3)	
H2A	0.6714	1.0761	0.6836	0.039*	
H2B	0.6302	1.1090	0.7160	0.039*	
C3	0.68086 (6)	0.6183 (2)	0.70644 (9)	0.0311 (3)	
H3A	0.6928	0.4816	0.7438	0.037*	
H3B	0.7140	0.6601	0.7037	0.037*	
C4	0.62582 (6)	0.5677 (2)	0.61410 (9)	0.0317 (3)	
H4A	0.6352	0.4494	0.5868	0.038*	
H4B	0.5941	0.5090	0.6177	0.038*	
C5	0.75644 (6)	0.7311 (2)	0.88472 (8)	0.0290 (3)	
H5	0.7586	0.5880	0.8640	0.035*	
C6	0.80153 (6)	0.7948 (2)	0.97695 (9)	0.0282 (3)	
C7	0.80100 (6)	1.0039 (2)	1.01127 (9)	0.0317 (3)	
H7	0.7706	1.1101	0.9737	0.038*	
C8	0.84416 (6)	1.0572 (3)	1.09893 (9)	0.0336 (3)	
H8	0.8438	1.1991	1.1221	0.040*	
C9	0.88821 (6)	0.8997 (3)	1.15268 (9)	0.0326 (3)	
C10	0.89029 (6)	0.6922 (3)	1.12129 (9)	0.0344 (3)	
H10	0.9208	0.5867	1.1594	0.041*	
C11	0.84702 (6)	0.6411 (2)	1.03298 (9)	0.0330 (3)	
H11	0.8482	0.4998	1.0101	0.040*	
C12	0.55353 (7)	0.7136 (3)	0.46826 (9)	0.0387 (4)	
H12A	0.5211	0.6537	0.4689	0.058*	
H12B	0.5654	0.6002	0.4436	0.058*	
H12C	0.5400	0.8502	0.4317	0.058*	

### Atomic displacement parameters (Å^2^)

**Table:**

	*U*^11^	*U*^22^	*U*^33^	*U*^12^	*U*^13^	*U*^23^
O1	0.0527 (8)	0.0698 (9)	0.0412 (7)	0.0008 (6)	0.0132 (6)	−0.0240 (6)
O2	0.0326 (6)	0.0792 (9)	0.0295 (6)	0.0096 (6)	0.0123 (5)	0.0014 (6)
N1	0.0278 (6)	0.0347 (6)	0.0256 (6)	−0.0026 (5)	0.0146 (5)	−0.0031 (5)
N2	0.0279 (6)	0.0289 (6)	0.0245 (6)	−0.0001 (4)	0.0138 (5)	−0.0035 (4)
N3	0.0278 (6)	0.0326 (6)	0.0257 (6)	−0.0038 (5)	0.0154 (5)	−0.0042 (4)
N4	0.0280 (6)	0.0624 (9)	0.0287 (6)	−0.0011 (6)	0.0159 (6)	−0.0066 (6)
C1	0.0290 (7)	0.0327 (7)	0.0301 (7)	0.0031 (5)	0.0140 (6)	−0.0005 (5)
C2	0.0335 (7)	0.0274 (7)	0.0310 (7)	0.0027 (5)	0.0156 (6)	−0.0022 (6)
C3	0.0312 (7)	0.0286 (7)	0.0284 (7)	0.0021 (5)	0.0150 (6)	−0.0031 (5)
C4	0.0334 (7)	0.0291 (7)	0.0302 (7)	−0.0029 (5)	0.0177 (6)	−0.0061 (5)
C5	0.0291 (7)	0.0304 (7)	0.0290 (7)	−0.0023 (5)	0.0181 (6)	−0.0026 (5)
C6	0.0271 (7)	0.0331 (7)	0.0278 (7)	−0.0036 (5)	0.0183 (6)	−0.0010 (5)
C7	0.0291 (7)	0.0354 (7)	0.0289 (7)	0.0009 (6)	0.0163 (6)	−0.0002 (6)
C8	0.0325 (7)	0.0365 (8)	0.0330 (7)	−0.0035 (6)	0.0202 (6)	−0.0065 (6)
C9	0.0261 (7)	0.0467 (8)	0.0264 (7)	−0.0050 (6)	0.0165 (6)	−0.0041 (6)
C10	0.0282 (7)	0.0437 (8)	0.0295 (7)	0.0037 (6)	0.0161 (6)	0.0037 (6)
C11	0.0325 (7)	0.0341 (7)	0.0334 (7)	0.0004 (6)	0.0201 (6)	−0.0018 (6)
C12	0.0333 (8)	0.0498 (9)	0.0265 (7)	−0.0039 (6)	0.0142 (6)	−0.0052 (6)

### Geometric parameters (Å, º)

**Table:**

O1—N4	1.2253 (19)	C4—H4A	0.9900
O2—N4	1.2219 (18)	C4—H4B	0.9900
N1—C1	1.4586 (17)	C5—H5	0.9500
N1—C4	1.4546 (18)	C5—C6	1.4598 (19)
N1—C12	1.4608 (17)	C6—C7	1.401 (2)
N2—N3	1.3682 (15)	C6—C11	1.400 (2)
N2—C2	1.4639 (17)	C7—H7	0.9500
N2—C3	1.4580 (16)	C7—C8	1.379 (2)
N3—C5	1.2889 (18)	C8—H8	0.9500
N4—C9	1.4657 (18)	C8—C9	1.388 (2)
C1—H1A	0.9900	C9—C10	1.379 (2)
C1—H1B	0.9900	C10—H10	0.9500
C1—C2	1.5137 (19)	C10—C11	1.384 (2)
C2—H2A	0.9900	C11—H11	0.9500
C2—H2B	0.9900	C12—H12A	0.9800
C3—H3A	0.9900	C12—H12B	0.9800
C3—H3B	0.9900	C12—H12C	0.9800
C3—C4	1.5122 (18)		
			
C1—N1—C12	110.78 (11)	C3—C4—H4A	109.4
C4—N1—C1	108.16 (10)	C3—C4—H4B	109.4
C4—N1—C12	110.55 (11)	H4A—C4—H4B	108.0
N3—N2—C2	110.17 (10)	N3—C5—H5	119.7
N3—N2—C3	119.30 (10)	N3—C5—C6	120.54 (13)
C3—N2—C2	113.80 (10)	C6—C5—H5	119.7
C5—N3—N2	120.39 (12)	C7—C6—C5	122.44 (13)
O1—N4—C9	117.78 (14)	C11—C6—C5	118.67 (13)
O2—N4—O1	123.67 (13)	C11—C6—C7	118.89 (13)
O2—N4—C9	118.55 (14)	C6—C7—H7	119.6
N1—C1—H1A	109.7	C8—C7—C6	120.72 (14)
N1—C1—H1B	109.7	C8—C7—H7	119.6
N1—C1—C2	109.86 (11)	C7—C8—H8	120.6
H1A—C1—H1B	108.2	C7—C8—C9	118.76 (13)
C2—C1—H1A	109.7	C9—C8—H8	120.6
C2—C1—H1B	109.7	C8—C9—N4	118.83 (13)
N2—C2—C1	111.02 (11)	C10—C9—N4	118.94 (13)
N2—C2—H2A	109.4	C10—C9—C8	122.22 (13)
N2—C2—H2B	109.4	C9—C10—H10	120.7
C1—C2—H2A	109.4	C9—C10—C11	118.56 (13)
C1—C2—H2B	109.4	C11—C10—H10	120.7
H2A—C2—H2B	108.0	C6—C11—H11	119.6
N2—C3—H3A	109.5	C10—C11—C6	120.84 (13)
N2—C3—H3B	109.5	C10—C11—H11	119.6
N2—C3—C4	110.69 (11)	N1—C12—H12A	109.5
H3A—C3—H3B	108.1	N1—C12—H12B	109.5
C4—C3—H3A	109.5	N1—C12—H12C	109.5
C4—C3—H3B	109.5	H12A—C12—H12B	109.5
N1—C4—C3	111.29 (11)	H12A—C12—H12C	109.5
N1—C4—H4A	109.4	H12B—C12—H12C	109.5
N1—C4—H4B	109.4		
			
O1—N4—C9—C8	−7.5 (2)	C3—N2—N3—C5	−21.56 (18)
O1—N4—C9—C10	171.92 (14)	C3—N2—C2—C1	50.72 (15)
O2—N4—C9—C8	172.30 (13)	C4—N1—C1—C2	62.44 (14)
O2—N4—C9—C10	−8.2 (2)	C5—C6—C7—C8	179.56 (12)
N1—C1—C2—N2	−56.86 (15)	C5—C6—C11—C10	−179.02 (12)
N2—N3—C5—C6	−176.24 (11)	C6—C7—C8—C9	0.0 (2)
N2—C3—C4—N1	55.30 (15)	C7—C6—C11—C10	1.1 (2)
N3—N2—C2—C1	−172.24 (11)	C7—C8—C9—N4	179.59 (12)
N3—N2—C3—C4	177.74 (11)	C7—C8—C9—C10	0.1 (2)
N3—C5—C6—C7	−0.5 (2)	C8—C9—C10—C11	0.4 (2)
N3—C5—C6—C11	179.62 (12)	C9—C10—C11—C6	−1.0 (2)
N4—C9—C10—C11	−179.07 (12)	C11—C6—C7—C8	−0.6 (2)
C1—N1—C4—C3	−62.15 (14)	C12—N1—C1—C2	−176.25 (11)
C2—N2—N3—C5	−155.91 (12)	C12—N1—C4—C3	176.40 (11)
C2—N2—C3—C4	−49.44 (15)		

### Hydrogen-bond geometry (Å, º)

**Table:**

*D*—H···*A*	*D*—H	H···*A*	*D*···*A*	*D*—H···*A*
C2—H2*B*···O1^i^	0.99	2.47	3.4052 (19)	158

Symmetry code: (i) −*x*+3/2, −*y*+5/2, −*z*+2.

## References

[bb1] Agilent (2012). *CrysAlis PRO* and *CrysAlis RED* Agilent Technologies, Yarnton, England.

[bb2] Allen, F. H., Kennard, O., Watson, D. G., Brammer, L., Orpen, A. G. & Taylor, R. (1987). *J. Chem. Soc. Perkin Trans. 2*, pp. S1–19.

[bb3] Bogatcheva, E., Hanrahan, C., Nikonenko, B., Samala, R., Chen, P., Gearhart, J., Barbosa, F., Einck, L., Nacy, C. A. & Protopopova, M. (2006). *J. Med. Chem.* **49**, 3045–3048. 10.1021/jm050948+ PMC4869334 16722620

[bb4] Brockunier, L. L., He, J., Colwell, L. F. Jr, Habulihaz, B., He, H., Leiting, B., Lyons, K. A., Marsilio, F., Patel, R. A., Teffera, Y., Wu, J. K., Thornberry, N. A., Weber, A. E. & Parmee, E. R. (2004). *Bioorg. Med. Chem. Lett.* **14**, 4763–4766. 10.1016/j.bmcl.2004.06.065 15324904

[bb5] Cai, J.-L., Lu, Y.-H., Gan, L.-L. & Zhou, C.-H. (2009). *Chin. J. Antibiot.* **34**, 454–462.

[bb6] Capuano, B., Crosby, I. T., Lloyd, E. J. & Taylor, D. A. (2002). *Aust. J. Chem.* **55**, 565–576.

[bb7] Choudhary, P., Kumar, R. & Verma, K. (2006). *Bioorg. Med. Chem.* **14**, 1819–1826. 10.1016/j.bmc.2005.10.032 16289939

[bb8] Contreras, J. M., Parrot, I., Sippl, W., Rival, Y. M. & Wermuth, C. G. (2001). *J. Med. Chem.* **44**, 2707–2718. 10.1021/jm001088u 11495583

[bb9] Cremer, D. & Pople, J. A. (1975). *J. Am. Chem. Soc.* **97**, 1354–1358.

[bb10] Dolomanov, O. V., Bourhis, L. J., Gildea, R. J., Howard, J. A. K. & Puschmann, H. (2009). *J. Appl. Cryst.* **42**, 339–341.

[bb11] Elliott, S. (2011). *Drug Test Anal.* **3**, 430–438 10.1002/dta.307 21744514

[bb12] Guo, M.-L. (2007). *Acta Cryst.* E**63**, o1788–o1789.

[bb13] Kharb, R., Bansal, K. & Sharma, A. K. (2012). *Pharma Chem.* **4**, 2470–2488.

[bb14] Ming-Lin, G. & You-Nong, Q. (2007). *Acta Cryst.* E**63**, o4641.

[bb15] Palatinus, L. & Chapuis, G. (2007). *J. Appl. Cryst.* **40**, 786–790.

[bb16] Savaliya, M. D., Dobaria, J. G. & Purohit, D. M. (2010). *An Indian J.* **6**, 267–271.

[bb17] Sheldrick, G. M. (2008). *Acta Cryst.* A**64**, 112–122. 10.1107/S0108767307043930 18156677

[bb18] Upadhayaya, P. S., Sinha, N. & Jain, S. (2004). *Bioorg. Med. Chem.* **12**, 2225–2238. 10.1016/j.bmc.2004.02.014 15080922

[bb19] Xu, R.-B., Xu, X.-Y., Wang, D.-Q., Yang, X.-J. & Li, S. (2009). *Acta Cryst.* E**65**, o2997. 10.1107/S1600536809045619 PMC2972073 21578737

[bb20] Xu, R.-B., Zhang, N., Zhou, H.-Y., Yang, S. P., Li, Y.-Y., Shi, D.-H., Ma, W.-X. & Xu, X.-Y. (2012). *J. Chem. Crystallogr.* **42**, 928–932.

[bb21] Zhou, L.-N., Yan, L., Zhou, H.-L., Yang, Q.-F. & Hu, Q.-L. (2011). *Acta Cryst.* E**67**, o100. 10.1107/S1600536810051135 PMC3050162 21522614

